# Marine Collagen-Based Bioink for 3D Bioprinting of a Bilayered Skin Model

**DOI:** 10.3390/pharmaceutics15051331

**Published:** 2023-04-24

**Authors:** Aida Cavallo, Tamer Al Kayal, Angelica Mero, Andrea Mezzetta, Anissa Pisani, Ilenia Foffa, Cecilia Vecoli, Marianna Buscemi, Lorenzo Guazzelli, Giorgio Soldani, Paola Losi

**Affiliations:** 1 Institute of Clinical Physiology, CNR, 54100 Massa, Italy; 2 Health Science Interdisciplinary Center, Scuola Superiore Sant’Anna, 56127 Pisa, Italy; 3 Department of Pharmacy, University of Pisa, 56126 Pisa, Italy

**Keywords:** marine collagen, bioink, 3D bioprinting, alginate, skin model

## Abstract

Marine organisms (i.e., fish, jellyfish, sponges or seaweeds) represent an abundant and eco-friendly source of collagen. Marine collagen, compared to mammalian collagen, can be easily extracted, is water-soluble, avoids transmissible diseases and owns anti-microbial activities. Recent studies have reported marine collagen as a suitable biomaterial for skin tissue regeneration. The aim of this work was to investigate, for the first time, marine collagen from basa fish skin for the development of a bioink for extrusion 3D bioprinting of a bilayered skin model. The bioinks were obtained by mixing semi-crosslinked alginate with 10 and 20 mg/mL of collagen. The bioinks were characterised by evaluating the printability in terms of homogeneity, spreading ratio, shape fidelity and rheological properties. Morphology, degradation rate, swelling properties and antibacterial activity were also evaluated. The alginate-based bioink containing 20 mg/mL of marine collagen was selected for 3D bioprinting of skin-like constructs with human fibroblasts and keratinocytes. The bioprinted constructs showed a homogeneous distribution of viable and proliferating cells at days 1, 7 and 14 of culture evaluated by qualitative (live/dead) and qualitative (XTT) assays, and histological (H&E) and gene expression analysis. In conclusion, marine collagen can be successfully used to formulate a bioink for 3D bioprinting. In particular, the obtained bioink can be printed in 3D structures and is able to support fibroblasts and keratinocytes viability and proliferation.

## 1. Introduction

In recent years, three-dimensional (3D) bioprinting has gained much attention in the field of tissue engineering due to the possibility of fabricating complex structures using biopolymers, bioactive molecules and live cells to regenerate defected tissues [1]. In 3D bioprinting, using an automated device for the layer-by-layer deposition of biomaterial and cell simultaneously enables improved precision and customisation compared to conventional tissue engineering methods [2]. The extrusion-based bioprinting, the most used of all bioprinting technologies, is based on a pneumatic pressure or a mechanical force to move a piston or a screw to extrude a cell-laden hydrogel known as bioink. In literature, there are many studies focused on bioink development because it is a key component of the 3D bioprinting process [3]. In particular, hydrogels are suitable for bioink preparation because they are characterised by a high content of water, biocompatibility and controllable mechanical properties providing a proper environment for living cells [4]. In many cases, the combination of different hydrogels is useful to obtain a printable bioink. Mammalian collagen is one of the most popular biomaterials in tissue engineering because it is the most abundant protein of the extracellular matrix of biological tissue and it is characterised by high biocompatibility and low immunogenicity [5]. However, collagen extraction from mammals is associated with the risk of triggering an immune reaction and transferring zoonosis [6]. Moreover, mammal-derived collagen purification is difficult and expensive while the use of bovine and porcine collagen is prohibited due to religious beliefs in Hinduism, Islamic and Jewish cultures, respectively [7]. In recent years, attention has been focused on marine organisms (i.e., fish, jellyfish, sponges or seaweeds) as a novel, safe and abundant source of collagen. Marine collagen, compared to mammalian collagen, can be easily extracted, is water-soluble, is a lower threat of transmissible diseases, has anti-microbial activities and has better chemical and physical durability. In addition, every year, the fish processing industry produces large amounts of discards (e.g., skin, bones, fins, heads, guts and scales) [8,9,10]. Thus, collagen extraction from fish by-products makes marine collagen eco-friendly and attractive in terms of profitability [11]. Considering the advantages mentioned above, the use of marine collagen in health-related fields is rapidly growing. In particular, marine collagen is used in food and nutraceuticals, cosmetics and nutricosmetics, weight management and glycaemic control [12].

With focus on tissue regeneration, many studies have reported the marine collagen extracted from fish skin as suitable biomaterial for skin tissue regeneration and wound-healing applications [13]. In particular, Pal et al. demonstrated that a marine collagen sponge, fabricated by the freeze-drying method, supported the growth and proliferation of primary human keratinocytes and fibroblasts in a 3D co-culture [14]. Recent studies exploit the use of marine extract as biomaterial for bioink preparation [15,16,17,18,19] and only a few of them are focused on a marine collagen bioink development [17,18]. 

However, standalone collagen solutions have difficulty being printed; for this reason, the strategy to add a printable biomaterial was considered. In particular, sodium alginate is another biomaterial of marine origin that has been deeply investigated in the tissue engineering field due to its low cost and good properties in terms of biocompatibility, biostability and printability, but it is not able to support cell growth and proliferation due to the lack of cell adhesion sites [20].

Further, during the last years, the development of the in vitro skin model has received great attention as alternative to in vivo or ex vivo animal skin experiments. In the literature, different 3D skin models, biofabricated using several techniques, are described [21,22,23].

The aim of this study was to investigate the marine collagen from basa fish skin for the development of a bioink for 3D bioprinting of a full-thickness skin substitute useful in vitro as a model according to the “3R principles”. To the best of our knowledge, this is the first study on the marine-collagen-based bioink development with human fibroblasts and keratinocytes for skin bioprinting applications. The obtained marine collagen was added to sodium alginate in two different concentrations and the physical-chemical, mechanical, biological and antimicrobial properties were evaluated to define the formulation with the best properties before skin substitute bioprinting.

## 2. Materials and Methods

### 2.1. Preparation of Marine Collagen Bioink

The collagen-alginate bioink was prepared by dissolving the sterile marine collagen powder from basa fish skin in RPMI 1640 medium (10 and 20 mg/mL) for 2 h at room temperature in agitation. Then, a sterile alginate powder (Merck KGaA, Darmstadt, Germany) was added to enrich the final concentration of 8%, and the collagen-alginate solution was mixed at a volumetric ratio of 25:9 with sterile 100 mM CaCl_2_ solution for 3 h at room temperature in agitation. A standalone alginate bioink was prepared as reference material. The composition of each bioink formulation is reported in Table 1. After printing, the bioinks were crosslinked by submersion in 50 mM of CaCl_2_ for 5 min. 

### 2.2. Bioink Homogeneity Evaluation

**Figure 1:**
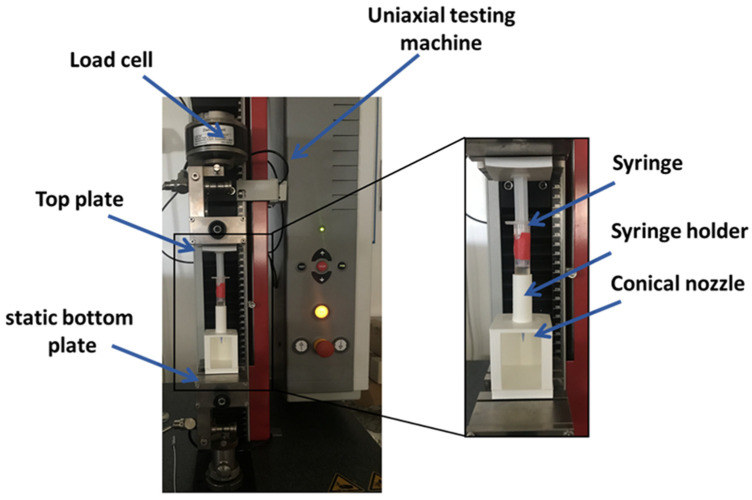
Setup for bioink homogeneity assessment composed by (i) uni-axial testing machine, (ii) custom 3D-printed syringe holder and (iii) syringe loaded with bioink and equipped with 22 G conical nozzle.

A dedicated setup (Figure 1) was implemented to measure the extrusion force that occurs during the 3D bioprinting process. A constant extrusion force indicates the homogeneity of bioink solutions [24]. The setup is composed by a uni-axial testing machine (Z 1.0, ZwickRoell GmbH, Ulm, Germany) and by a holder to keep the syringe in a vertical position. The custom components of setup were designed using free CAD software and fabricated in polylactide (PLA) by 3D printing (SharebotQ, Sharebot, Lecco, Italy).

The syringe loaded with bioink and equipped with a conical nozzle of 22 G (0.41 mm as internal diameter) was mounted into the holder on the static bottom plate of the mechanical testing machine. The syringe plunger was connected to the top plate of the machine that was set in a compression mode using the testExpert software (ZwickRoell GmbH, Ulm, Germany). The test was performed with 0.25 mm/s as the speed displacement of the top plate to extrude the bioink and the extrusion force was measured.

### 2.3. Rheological Properties

The rheological properties of collagen-based bioink were measured using a modular compact rheometer (MCR 302, Anton Paar, Turin, Italy) equipped with a plate–plate geometry (Ø = 5 cm) and a protective hood. All experiments were carried out at constant temperatures (25 °C and 37 °C) controlled by a water-cooled Peltier system (H-PTD200, Anton Paar). To optimise printing parameters, rotational tests (shear rate sweep) and oscillatory tests (amplitude sweep and frequency sweep) were performed [25]. At first, the shear thinning behaviour was ascertained by conducting flow sweep experiments, varying the shear rate from 0.1 to 1000 s^−1^. Fifty data points were collected by the rheometer every 20 s. Then, the storage modulus G’ and the loss modulus G” were determined by oscillatory experiments. Amplitude sweeps were performed between 0.1 and 500%, applying a constant frequency of 1 Hz while frequency sweeps were carried out over a range of oscillation frequencies between 100 and 0.1 rad/s at 0.1% of shear strain.

### 2.4. Printability Assessment

The printability is a fundamental property of bioink and was assessed in terms of filament collapse, spreading ratio, shape fidelity, inter-filament line spacing and printable angles.

**Figure 2:**
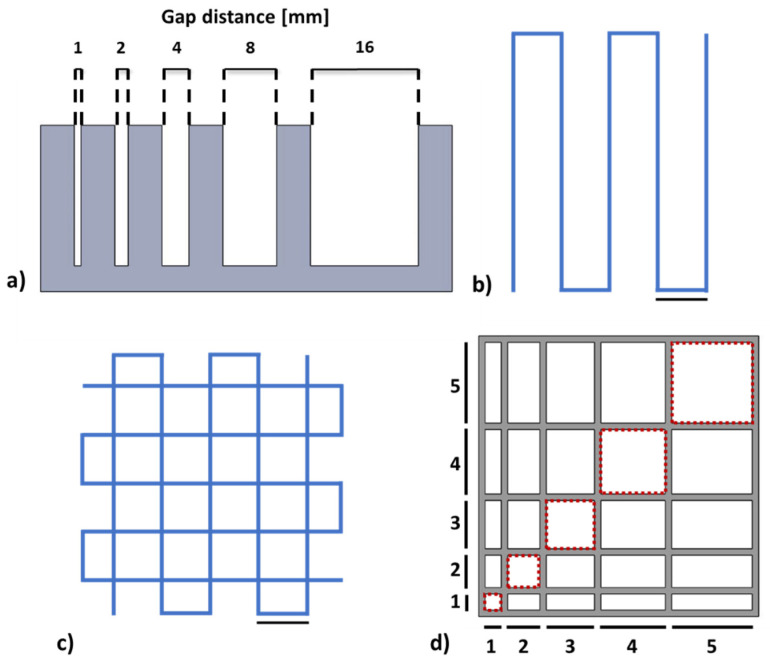
(**a**) CAD model of custom platform for filament collapse test performing; patterns for assessment of (**b**) spreading ratio; (**c**) shape fidelity and (**d**) inter-filament line spacing (the reported measures are in mm); scale bar 5 mm.

The filament collapse test was performed according to the literature to assess the mid-span deflection of a suspended filament of bioink [26]. To perform the test, a platform with 6 pillars with different gap distances (1, 2, 4, 8 and 16 mm shown in Figure 2) was designed using free CAD software and fabricated in PLA using a SharebotQ 3D printer. The bioink was loaded into the cartridge with a conical nozzle of 22G and deposited using the extrusion printhead of BIO X (Cellink, Gothenburg, Sweden) after calibration on the first platform pillar. The printing speed was set at 7 mm/s while the extrusion pressure was set at 25 kPa for ALG, and at 28 and 30 kPa for COL-ALG-10 and COL-ALG-20, respectively. The photos captured 20 s after printing were analysed using ImageJ software (NCBI) to measure the deflection angle Ɵ.

The spreading ratio (SR) is calculated as the ratio between the extruded filament width and the nozzle diameter while the shape fidelity (*Pr*) according to the following equation [27]:

$$Pr=\frac{L^{2}}{16A}$$

where *L* is the interconnected pore perimeter and *A* is the area. Two dedicated patterns (Figure 2a,b) were designed in CAD software for the printability evaluation. The CAD model was converted into an .STL file compatible with an open-source slicing software (Slic3r) useful for generating a G-CODE, a file format compatible with the 3D bioprinter. The printing speed was set at 7 mm/s while the pneumatic pressure value was adjusted before stating the bioprinting process and set to 18 kPa for ALG and to 20 kPa and 23 kPa for COL-ALG-10 and COL-ALG-20, respectively. The set pressure values enable to obtain filament-like extrusion for each bioinks. Three patterns were printed to evaluate SR and three for *Pr* for each bioink formulation. Pictures of printed patterns were captured and analysed using ImageJ software to calculate the SR and *Pr*.

A pattern with a linear increase in line spacing in both X and Y directions was designed (Figure 2d) in order to assess the inter-filament line spacing. The patterns were printed and images were captured under a stereomicroscope (Zeiss Axio Zoom.V16, Carl Zeiss, Oberkochen, Germany) immediately after printing to assess the effective line spacing dimensions.

Finally, in order to assess the printable angles and therefore the bioink printing versatility, CAD designs of a circle, a square and a triangle were made and samples were 3D printed [28]. They were observed under the stereomicroscope and the angles were calculated using Image J.

### 2.5. Mechanical Properties

**Figure 3:**
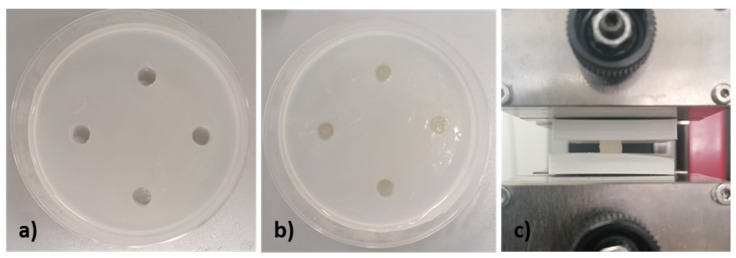
(**a**) Agarose mould and (**b**) crosslinked samples for (**c**) compression mechanical test performing.

To investigate the mechanical properties, three samples with a diameter of 8 mm and height of 5 mm were prepared by casting for each bioink. Briefly, 3% (*w/v*) of agarose (Merck KGaA) was dissolved in 50 mM CaCl_2_ solution at 80 °C in agitation, then was immediately poured into a Petri dish and cooled down at room temperature. The agarose gel was used as a mould in particular, holes with the same dimensions of sample were cut within the gel (Figure 3a), filled with bioink (Figure 3b) and left for 2 h at 37 °C to crosslink. Mechanical compression tests (Figure 3c) were performed using the mechanical testing machine equipped with a 10 N load cell, at 1 mm/min and with a maximum displacement of 60%. For each sample, the compressive force and displacement were collected using the TestXpert II software to obtain the stress–strain graph.

### 2.6. Morphological Properties

The bioink morphology was evaluated in order to assess the microporosity of the bioprinted structures. Samples of crosslinked bioinks were freeze-dried for 24 h, sputtered with gold nanoparticles and then observed at scanning electron microscopy (FlexSEM 1000, Hitachi, Tokyo, Japan). Images were captured at an accelerating voltage of 5 kV, with the spot intensity equal to 10 and 10-mm working distance.

### 2.7. Physical Properties

The physical properties such as the degradation rate and the swelling index were evaluated for each bioinks on bioprinted constructs [25]. In particular, circular constructs with an 8 mm diameter and a 2 mm height were printed. 

The degradation analysis was performed on three cross-linked constructs for each bioink. Briefly, the samples were weighted (*Wo*) and then incubated at 37 °C submerged in a culture medium to mimic the standard cell conditions. On days 7, 10, 14 and 21, the excess of medium was removed and samples weighed (*Wt*). The degradation rate was calculated according to the following equation:

$$degradation\;rate\;(\%)=\frac{Wt-Wo}{Wo}\times 100$$

For the evaluation of swelling, samples were freeze-dried for 24 h, weighed (*Wd*) and incubated in PBS. At 2, 4, 6 and 24 h later, samples were weighed (*Wh*) and the swelling index (*SI*) was calculated according to the following equation:

$$SI=\frac{Wh}{Wd}$$

### 2.8. In Vitro Antibacterial Activity of Bioinks

*Escherichia coli* (Gram-negative bacteria) and *Staphylococcus aureus* (Gram-positive bacteria) were selected for the evaluation of antimicrobial activities of marine collagen solutions and of collagen-based 3D printed constructs by inhibition zone assay [29].

Briefly, sterile circular filter papers (8 mm in diameter) soaked with 100 µL of 10 and 20 mg/mL marine collagen solution and 3D constructs (circular shape with a volume of about 100 µL) printed using COL-ALG-10 and COL-ALG-20 bioinks were placed on Mueller–Hinton agar with a bacterial inoculation density of 1 × 10^6^ and 1 × 10^7^ colony-forming unit (CFU) of both bacteria. After 24 h of incubation at 37 °C, the average diameter of the inhibiting zone around the samples was measured using image J software.

### 2.9. 3D Bioprinting

Normal Human Dermal Fibroblasts (NHDF, Promo Cell) and HaCaT keratinocytes (Istituto Zooprofilattico Sperimentale della Lombardia e dell’Emilia Romagna “Bruno Ubertini”) were cultured in high-glucose DMEM supplemented with 10% of FBS, 1% of glutamine and 1% of penicillin and streptomycin at 37 °C and 5% CO_2_.

For the skin substitute bioprinting, NHDF and HaCaT cells were harvested using 1% trypsin-EDTA (Merck KGaA), pelleted, resuspended in 100 µL of medium and added to COL-ALG-20 bioink to have a final concentration of 4 × 10^6^ and of 6 × 10^6^ cell/mL, respectively. Each cell-laden bioink was loaded into a different cartridge equipped with a 22 G nozzle. The extrusion pressure and printing rate were set to 22 kPa and 7 mm/s, respectively, for the cell-laden constructs’ 3D bioprinting. The constructs are composed of four layers of fibroblasts and two layers of keratinocytes. After printing, the samples were crosslinked by submersion in 50 mM CaCl_2_ for 5 min and incubated in complete medium. After 5 days of submerged culture, the air liquid interface (ALI) was performed, and the constructs were cultured for up to 14 days.

### 2.10. Cell Viability Assay

The live/dead assay was performed for a qualitative evaluation of cell viability. The test (CBA415, Merck KGaA) consists of selective stain: Calcein-AM to stain live cells, Propidium Iodide to stain dead cells and, in addition, Hoechst 33,342 to stain all cell nuclei. A cell-labelling approach was employed to distinguish between live and dead cells but also to identify cell morphology. Briefly, on days 1, 7 and 14 of culture, samples were washed twice using PBS solution and after 30 min of incubation in live/dead staining solution, the samples were washed with PBS solution. The cell viability/morphology was assessed by images randomly taken from constructs observed with the fluorescence stereomicroscope. In particular, the samples images were obtained using a Z-stack reconstruction (Zen Blue, Carl Zeiss) with 400 μm as the depth range.

### 2.11. Cell proliferation Assay

The XTT assay (Cell Proliferation kit II, Merck KGaA) was performed to quantify the cell viability at different time points and, therefore, to evaluate if the cells are proliferating within the 3D bioprinted constructs. The assay is based on the cleavage of the yellow tetrazolium salt XTT to form an orange formazan dye by metabolic active cells. The formazan dye formed is soluble in aqueous solution and is directly quantified using the microplate reader. The amount of orange formazan formed directly correlates to the number of living cells. Briefly, on days 1, 7 and 14 of culture, 250 µL of XTT solution was added to each well containing the printed sample in 500 µL of complete medium. After 4 h of incubation, the absorbance of the supernatant was measured at 450 and 620 nm using the microplate reader.

### 2.12. Gene Expression Analysis

The gene expression study was carried out at 0, 7 and 14 days to analyze the major components of the ECM synthesised by fibroblasts such as fibronectin, collagen I, collagen III, elastin [30] and cytokeratin CK6, considered a hallmark of keratinocytes hyperproliferation [31]. β-actin was used as reference gene. Total RNA was extracted from samples first mechanically broken by using a miRNeasy Mini Kit (Qiagen, Milan, Italy) tissue homogeniser according to the manufacturer’s recommendations. cDNA synthesis was produced with an iScript cDNA Synthesis kit (Bio-Rad, Hercules, CA, USA). RT-PCR was performed in 10 µL target volume using 1 µL cDNA, gene-specific forward and reverse primer, and 1× SYBR Green PCR Master mix (Bio-Rad, Milan, Italy). All RT-PCRs were performed using a 384-well CFX RT-PCR System (Bio-Rad). qRT-PCR analyses were run for each primer set in triplicates. The list of primer sequence is provided in Table S1.

### 2.13. Histological Analysis

On days 1, 7 and 14 of culture, 3D bioprinted constructs were fixed in 4% paraformaldehyde solution in 50 mM CaCl_2_ for 20 h. Samples were washed in H_2_O and dehydrated in ethanol series and paraffin embedded. A total of 7 µm sections were cut by the microtome (HM350S, Microm) and placed on Poly-L-Lysine-coated slides for Hematoxylin and Eosin (H&E) staining. 

### 2.14. Statistical Analysis

The results of a minimum of three replicates in three independent experiments were averaged and expressed as mean ± standard deviation. Statistical significance among the test and the control values (test executed immediately after printing) were determined by an unpaired t-student test and the values were considered significant at *p* ≤ 0.05.

## 3. Results

### 3.1. Bioinks Printability Assessment

**Figure 4:**
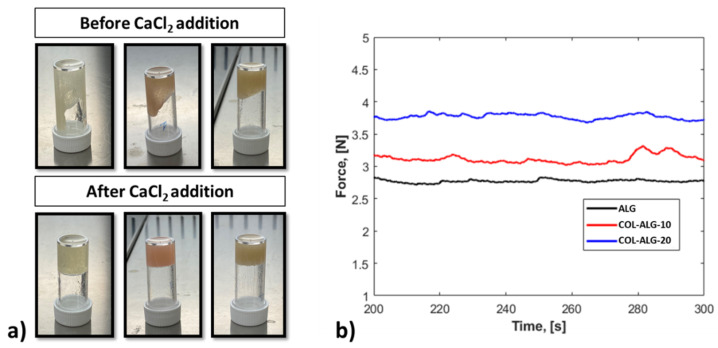
(**a**) Bioinks viscosity before and after CaCl_2_ addition to obtain a semi-crosslinked bioink for: ALG (left), COL-ALG-10 (middle) and COL-ALG-20 (right) bioinks; (**b**) Extrusion force measured for bioinks homogeneity assessment.

Standalone alginate as well as marine collagen and alginate-based solutions cannot be extruded, therefore a semi-crosslinking with 100 mM CaCl_2_ solution at volumetric ratio of 25:9 was used. The semi-crosslinked solutions result in a more viscous solution, as qualitatively shown in Figure 4a.

The homogeneity of each bioink formulation was investigated measuring the extrusion force in a dedicated setup able to simulate the 3D bioprinting extrusion process. The obtained results are shown in Figure 4b. In particular, if a constant displacement rate is imposed on the syringe plunger loaded with a homogeneous solution, the material uniformity would yield a constant extrusion force. According to this, the tested formulations are homogeneous, and the measured extrusion force (mean ± standard deviation) is equal to 2.7 ± 0.02 N for the ALG, 3.11 ± 0.07 N for COL-ALG-10 and 3.77 ± 0.04 N for COL-ALG-20. The extrusion force magnitude increases according to collagen concentration within the bioink.

**Figure 5:**
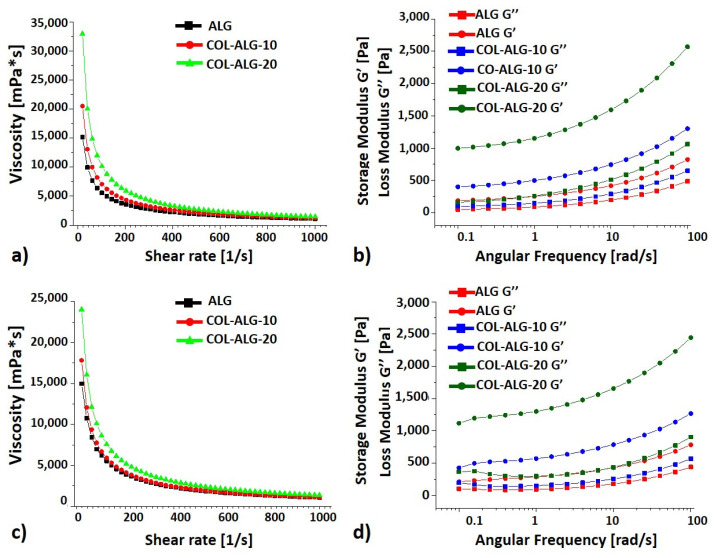
Rheological characterisation of ALG, COL-ALG-10 and COL-ALG-20 bioinks at (**a**,**b**) 25 °C; (**c**,**d**) 37 °C.

The rheological properties were analysed to investigate bioinks printability, the curves of viscosity vs. shear rate and of Storage (G’) and Loss (G’’) modulus vs angular frequency at 25 °C and 37 °C which represent standard printing condition and physiological cell temperature, respectively, are reported in Figure 5. As expected, the tested formulations showed decreasing viscosity with increasing shear rate at both testing temperatures, revealing that bioinks are non-Newtonian fluids with a shear thinning behaviour [32]. Bioinks viscosity increases concomitantly with collagen concentration, and at 37 °C all tested samples are characterised by lower viscosity compared with the samples tested at 25 °C. Moreover, marine collagen addition has also an impact on storage modulus (G’) and loss modulus (G’’). Both storage and loss modulus increase according to marine collagen concentration increasing and, in particular, the COL-ALG-20 is the formulation with much greater storage modulus than loss modulus at both testing temperatures.

**Figure 6:**
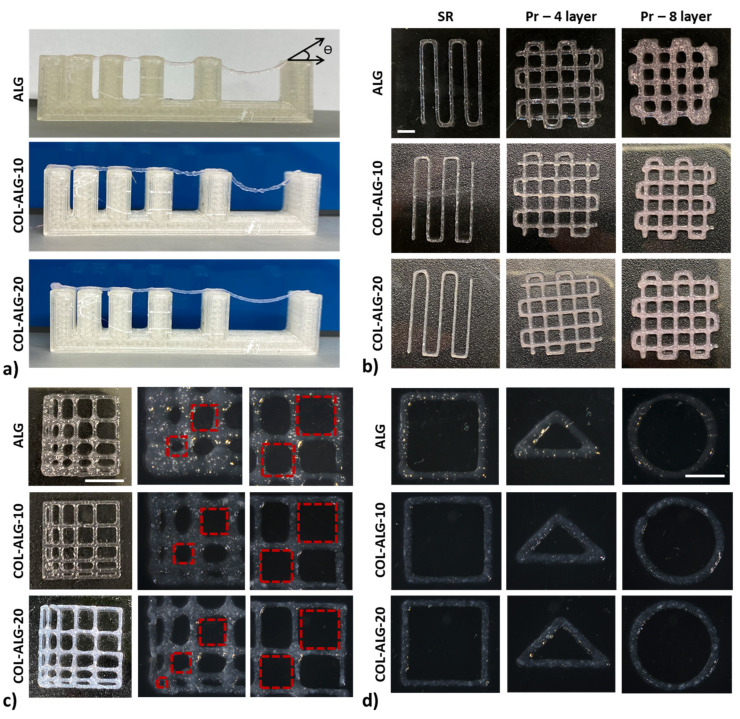
Representative images of 3D bioprinting: (**a**) filament on custom platform with six pillars at 1, 2, 4, 8 and 16 mm of gap distances; (**b**) patterns for the evaluation of spreading ratio (SR) and shape fidelity (*Pr*) at 4 and 8 layers; (**c**) patterns to assess the inter-filament line spacing (red dotted squares represent the ideal pattern) and (**d**) patterns to investigate printable angles.

The filament collapse test was performed using a custom platform with six pillars at gap distances of 1, 2, 4, 8 and 16 mm. In Figure 6a, the images captured after filament printing using each bioink formulation are reported. The ALG bioink is characterised by a deflection angle of 8° for a distance between pillars of 4 mm, while 50° and 55° were measured at gap distances of 8 and 16 mm, respectively. For the collagen-based bioinks, it is possible to appreciate a filament deflection only for the gap distances of 8 and 16 mm. In particular, the deflection angle is equal to 6° and 4° at 8 mm for COL-ALG-10 and COL-ALG-20, respectively, while is equal to 37° and 24° for COL-ALG-10 and COL-ALG-20, respectively, between the pillars with a distance of 16 mm. 

Representative images of bioprinted patterns using the collagen-based bioinks and the alginate bioink employed to calculate the spreading ratio and the shape fidelity are shown in Figure 6b. The ideal value for both calculated parameters is 1. As reported in Table 2, the spreading ratio values decrease according to the collagen concentration and the bioink COL-ALG-20 has a value nearest to the ideal one. The shape fidelity improves according to the marine collagen concentration increment but the shape fidelity evaluated at eight overlapped layers is worse when compared to four overlapped layers, also for the COL-ALG-20 bioink, that showed a proper shape fidelity between CAD model and 3D bioprinted construct.

The inter-filament line spacing was assessed to investigate the bioink versatility in macro holes bioprinting to improve media exchange. As shown in Figure 6c, for 1 mm of gap between filaments, a partial filament fusion was observed with a consequenting pores closure for ALG and COL-ALG-10 while, for 2 mm of gap, the partial filament fusion was observed only for the ALG bioink. For an increased gap between filaments, all formulations can be bioprinted without filament fusion. 

The bioink versatility in terms of different printable shape was assessed printing a square, a triangle and a circle corresponding to angles of 90°, 45° and 180° (Figure 6d). The proposed bioink formulations allowed the printing of the different shapes confirming the bioink versatility. 

### 3.2. Mechanical Properties

**Figure 7:**
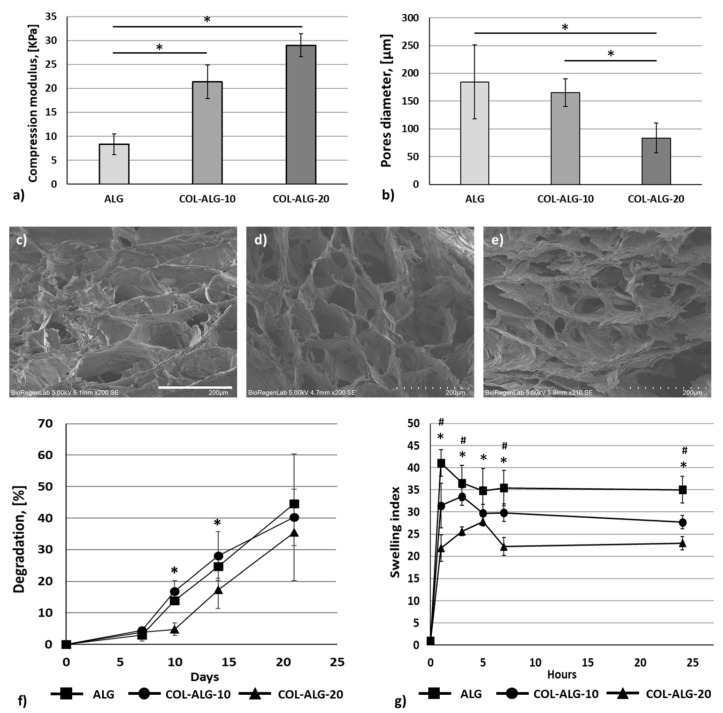
(**a**) Compression modulus, (**b**) pore diameter of crosslinked samples; SEM images of 3D bioprinted constructs: (**c**) alginate bioink ALG, (**d**) 10 mg/mL (COL-ALG-10) and (**e**) 20 mg/mL (COL-ALG-20) marine collagen-based bioink; scale bar 200 µm; (**f**) degradation and (**g**) swelling index of ALG, COL-ALG-10 and COL-ALG-20; # *p* < 0.05 COL-ALG-10 compared with ALG; * *p* < 0.05 COL-ALG-20 compared with ALG.

Compression tests were performed for the mechanical characterisation of the proposed bioink formulations. In Figure 7a, the compression modulus, calculated for each formulation considering the linear slope of stress–strain graphs, is reported. The compression modulus increases according to the marine collagen concentration and, in particular, there is a statistically significant (*p* < 0.05) increase of the stiffness of bioinks formulated with marine collagen compared to the standalone alginate bioink.

### 3.3. Morphological Properties

The morphological structure of bioprinted and crosslinked samples using all the proposed bioink formulations was investigated because the pore size is important for efficient mass transport and, therefore, for gas and nutrient exchange in cell-laden samples. The addition of collagen to the alginate solution leads to changes in the microstructure of the scaffold. The pores have a diameter of 185 ± 66 µm for ALG, and of 165 ± 25 µm and 83 ± 27 µm for COL-ALG-10 and COL-ALG-20, respectively, resulting in a statistically significant (*p* < 0.05) reduction of pore diameter for the COL-ALG-20 compared with ALG and COL-ALG-10 (Figure 7b). The images of the internal structure of samples are reported in Figure 7c–e. The interconnected micropores have a more defined shape with a smaller diameter as the concentration of collagen increases.

### 3.4. Physical Properties

The in vitro degradation rate was obtained after 21 days of 3D bioprinted and crosslinked samples submersion in complete culture medium. The sample weight was evaluated at days 7, 10, 14 and 21 of submersion. In Figure 7f, the percentage of weight loss calculated as the ratio between weight loss and the initial sample weight is reported. The ALG and COL-ALG-10 bioinks are characterised by similar degradation profiles with a weight lost at day 21 of 44 ± 20% and 40 ± 5%, respectively. The COL-ALG-20 bioink has a statistically significantly (*p* < 0.05) lower degradation profile with a mass loss of 35 ± 4% compared with ALG. 

The swelling index, defined as the ratio between the hydrated and dried weight of the sample, was calculated for the proposed bioink formulations and the results are reported in Figure 7g. The hydrated sample weight was measured at 1, 3, 5, 7 and 24 h of incubation in PBS. The swelling index is statistically significantly (*p* < 0.05) lower for both the collagen-based bioink compared with alginate bioink. In addition, the swelling index of COL-ALG-20 bioink is significantly lower than COL-ALG-10.

### 3.5. In Vitro Antibacterial Activity of Bioinks

**Figure 8:**
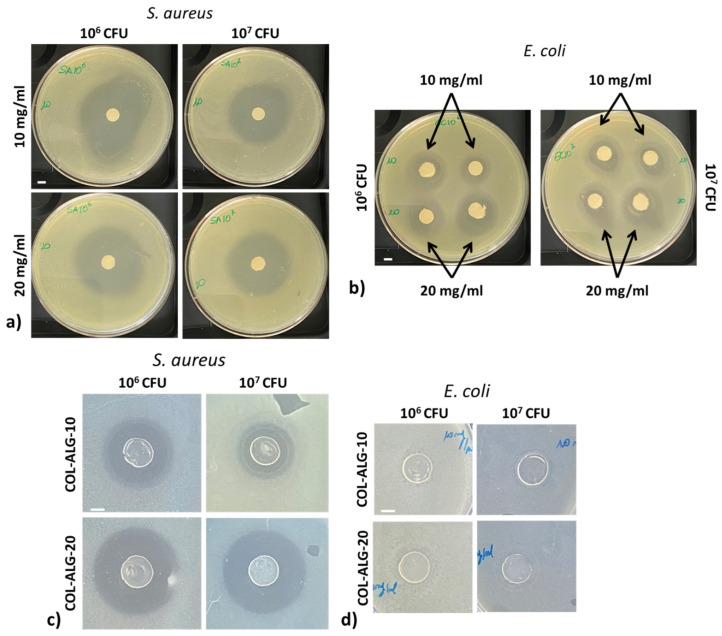
Inhibition zone assay performed for marine collagen solutions (10 and 20 mg/mL) and samples bioprinted using marine collagen-based bioinks (COL-ALG-10 and COL-ALG-20) against (**a**–**c**) *S. aureus* and (**b**–**d**) *E. coli;* scale bar 5mm.

Antimicrobial properties of marine collagen solutions and 3D bioprinted samples using marine collagen based bioinks were assessed by inhibition zone assay against 10^6^ e 10^7^ CFU of *S. aureus* and *E. coli*. The images of inhibition zones are reported in Figure 8, while the diameters of each zone are reported in Table 3. The diameter of the inhibition zone is directly proportional to the collagen concentration (i.e., the diameter is higher for the solution or bioink with the highest collagen concentration) and indirectly proportional to the CFU number (i.e., the diameter is lower for the highest bacteria CFU number). For the marine collagen solution, there is not a statistically significant difference in terms of inhibition zone diameter compared with the collagen concentration (10 and 20 mg/mL) or with the CFU number for both tested bacteria.

### 3.6. 3D Bioprinting

**Figure 9:**
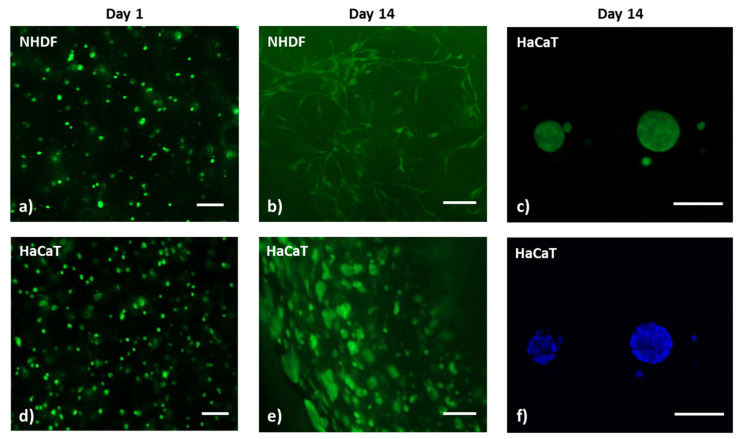
Calcein AM staining of 3D bioprinted constructs with (**a**,**b**) NHDF and (**d**–**e**) HaCaT cell; (**c**–**f**) Calcein AM (green) and Hoechst 33342 (blue) staining of HaCaT cell clusters in the 3D bioprinted constructs; scale bar 100 µm.

The skin substitute was bioprinted using the bioink formulation with 20 mg/mL of marine collagen (COL-ALG-20). The bioink was loaded with human fibroblasts (NHDF, 4 × 10^6^/mL) and with human keratinocytes (HaCaT, 5 × 10^6^/mL) for the dermal and epidermal layer bioprinting, respectively. The 3D construct was crosslinked by submersion in 50 mMCaCl_2_ for 5 min. The cell viability and distribution within the 3D bioprinted construct were preliminary investigated by live/dead staining (Figure 9) on days 1 and 14, as they are the most commonly evaluated time points. On day 14 of culture, the fibroblasts had spread extensively and had formed long fibrous shapes with continuous proliferation, meaning that most cells were long and fibrous (Figure 9a,b). HaCaT cells appear like round colonies of increased diameters which are composed of a variable number of cells (Figure 9d,e) at day 14 compared with day 1, as shown in Figure 9c–f. Moreover, a homogeneous cell distribution within the structure was observed. 

**Figure 10:**
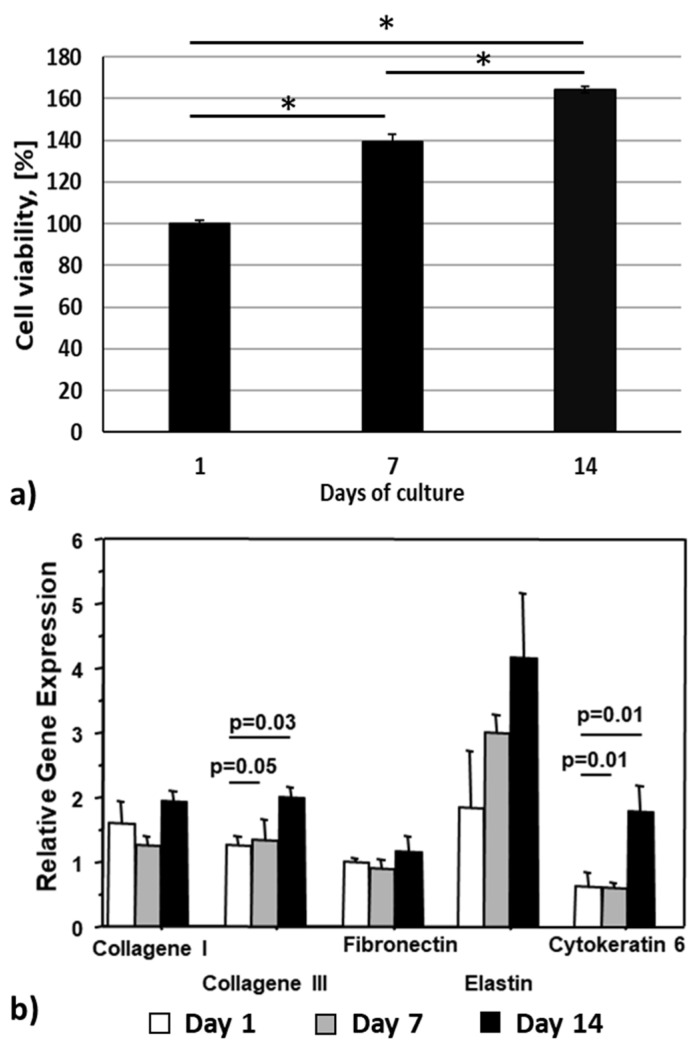
(**a**) XTT assay performed at day 1, 7 and 14 of in vitro culture, * *p* < 0.05 (cell viability assessed at day 0 was assumed as 100%); (**b**) relative gene expression at day 1, 7 and 14 of in vitro culture (b-actin was used as reference gene).

The cell viability was quantitatively evaluated performing the XTT assay (Figure 10a). The amount of metabolised XTT salt is directly proportional to the number of viable cells within the construct. The cell viability evaluated after printing was assumed as 100%. A cell viability of 139 ± 4% and 164 ± 1% was measured at days 7 and 14 of culture, respectively, demonstrating cell proliferation. 

As shown in Figure 10b, the skin substitute shows a positive expression of fibroblast and keratinocyte specific markers (day 1). On moving from day 7 to day 14, a statistically significant increase in the expression of collagen III, elastin and CK6 was found into the 3D printed constructs. The expression levels of fibronectin and collagen I gene were also higher in the 3D bioprinted construct at day 14 compared to day 7, although the difference did not reach the statistical significance.

**Figure 11:**
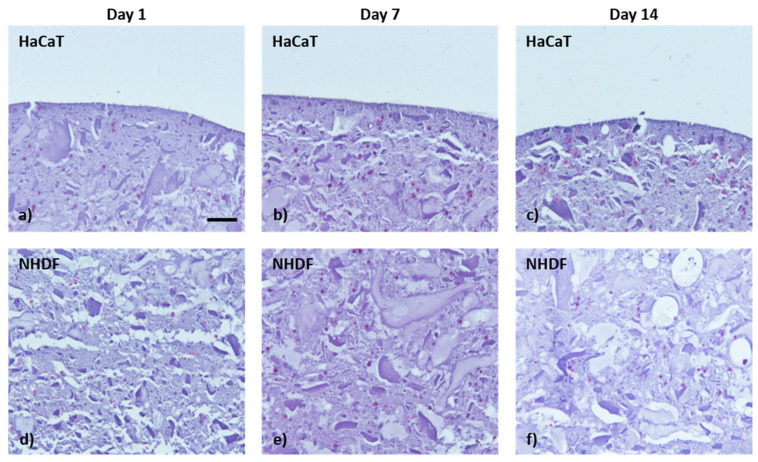
H&E staining on sample fixed at days 1, 7 and 14 of in vitro culture: (**a**–**c**) Epidermal layer with HaCaT cell and (**d**–**f**) dermal layer with NHDF cell. The pink dots represent cells. Scale bar 100 µm.

The histological analysis was performed to deeply investigate the cell distribution within the biofabricated bilayered structure. In Figure 11, the H&E staining of histological section of fixed samples at days 1, 7 and 14 of culture is reported. The cell-laden bioink enables to biofabricate a structure characterised by two distinct layers with fibroblasts on the bottom of the figure and keratinocytes on the top and with a homogeneous cell distribution within each layer. Moreover, the diameters of HaCaT keratinocyte clusters qualitatively increase at day 14 compared with the days 1 and 7, such as the number of NHDF cells being higher. 

## 4. Discussion

As a biofabrication technology, 3D bioprinting has emerged due to the possibility of living cells, biological chemicals and biomaterials’ contemporary deposition to mimic native tissue architecture [1]. Bioink is a key factor of the bioprinting process and many efforts of research are focused on novel bioink development to closer extracellular matrix mimicking. Discarded fish skin represents a valid alternative to mammals as a source for collagen extraction. Marine collagen has been investigated for applications in biomedical fields and, in particular, for wound-healing applications [33].

The aim of the present study Is to investigate marine collagen extracted from basa fish skin for the development of a novel marine collagen-based bioink for skin substitute bioprinting. Three different formulations, standalone alginate (ALG), 10 mg/mL and 20 mg/mL of marine collagen with alginate (COL-ALG-10 and COL-ALG-20) have been proposed and investigated.

Bovine and rat tail collagen powder requires acid solutions to be dissolved [34,35] and, therefore, solution pH neutralisation is necessary before cell addition. On the other hand, marine collagen powder has the great advantage of being water soluble; hence, in this study, it was dissolved in a culture medium and, as confirmed by the colour of all formulations, the proposed bioinks have a neutral pH and therefore neutralisation is not required [18].

The bioink sterility is a key aspect for the cell-laden construct biofabrication. In this study, the sterile formulation was prepared using sterile marine collagen, while sterile alginate powder was obtained by sterile filtration at low alginate concentration, followed by lyophilisation according to Lorson et al. [36]. The filtration method was also employed for the sterilisation of CaCl_2_. Moreover, all procedures were performed using sterile tools and under a laminar flow hood to maintain the sterility. Bioink printability is fundamental for successful bioprinting applications [32]. A printable bioink for pneumatic extrusion bioprinting is expected to be characterised in terms of a controllable formation of well-defined filament and the shape fidelity of deposited construct compared to the 3D model. Moreover, bioink printability is related to the precision of the bioprinting process, allowing a uniform cell distribution within the 3D constructs and avoiding the structure collapsing with layers fusion [37]. Therefore, the bioink formulation needs to be optimised by choosing the concentration of each component and checking the printability.

In this study, the printability was investigated in terms of solution homogeneity, rheological properties, filament collapse, spreading ratio, shape fidelity, inter-filament line spacing and angles printing. Proposed bioinks have been semi-crosslinked, adding small amounts of CaCl_2_ solution to the alginate or marine collagen–alginate solution to increase the viscosity and to have extrudable bioinks with a filament-like shape. Hence, the formulations were extruded, using a dedicated setup, resulting in homogeneous solutions. Despite the semi-crosslinking with CaCl_2_ being a rapid and poorly controlled gelation process [38], adding small amount of 100 mM CaCl_2_ to the solution allows the obtaining of homogeneous formulations. The measured values of the force represent the force required, during the bioprinting process, to obtain a filament-like extrusion. The force magnitude depends on the displacement rate, material viscosity and orifice diameter [24]. 

Regarding the rheological properties, a solution to be defined as bioink for extrusion-based bioprinting technology should have a shear thinning behaviour [32]. For the proposed formulations, viscosity increases concomitantly with collagen concentration increasing and this viscosity increase could lead to an improvement of printability [39]. According to data reported in the literature [40], collagen concentration also had an impact both on storage modulus (G’) and on loss modulus (G’’). Collagen bioink with a much greater storage modulus than loss modulus is suitable for direct extrusion bioprinting, and the COL-ALG-20 bioink has this characteristic. Moreover, bioinks’ viscosity correlates with extrusion force magnitude measured during bioinks’ homogeneity assessment; the highest extrusion force corresponds to the highest viscosity.

Furthermore, the marine collagen concentration affects the bioink printability, and the COL-ALG-20 bioink has a better printability compared with the other proposed formulations because it is characterised by lower bending angles in the filament collapse test, by spreading ratio and shape fidelity values nearest 1 and by 1 mm as lowest inter-filament line spacing. Hence, these results are in accordance with the evaluated rheological properties. The bioink COL-ALG-20 with the highest viscosity has also the best printability. In their study, Boonyagul et al. proposed a blend of fish scale gelatine and alginate for 3D bioprinting applications with a printing accuracy of about 95% [15]. Considering this value and the reported picture of bioprinted construct, the COL-ALG-20 proposed in this study is characterised by a better printability. Govindharaj et al. proposed a marine eel fish collagen and alginate bioink and reported the pictures of printed structure using the proposed formulation [18]. The collagen-based bioinks are characterised by a partial or total filament fusion for a inter filament line spacing of 1 or 2 mm, while the COL-ALG-20, proposed in the present study, can be printed with a higher accuracy. The COL-ALG-20 printability assessment is in line with results reported for a collagen-based bioink in terms of inter-filament line spacing by Yang et al. and by Somasekharan et al. using collagen and gelatine, blended with another extrudable biopolymer, respectively [25,41].

The mechanical properties of the bioprinted construct are important for simulating the surrounding matrix characteristics and are essential for cell spreading but also for construct handling [28]. The mechanical characterisation was performed after the crosslinking of biofabricated samples using each one of bioink formulations proposed. The marine collagen concentration increases the construct stiffness resulting in more robust and easily handled samples; however, all the formulations showed a compression modulus in the range of characteristics of a soft hydrogel for skin tissue engineering or soft tissue bioprinting described in the literature [30,42]. Yang et al., in their study on the development of a recombinant human collagen-based bioinks for the 3D bioprinting of a skin equivalent, reported a compression modulus of 22.6 kPa that is similar to the compression modulus of both collagen-based bioinks proposed [42]. 

Bioink after printing should be able to support cell viability and proliferation; therefore, an adequate exchange of nutrients, gasses and metabolic products should be supported by bioink microstructure. With this purpose, bioink’s morphology was investigated by SEM images acquisition. All samples showed an interconnected network of micropores which allows culture medium diffusion and, therefore, nutrients and gases to reach the cells. Bioprinted samples using COL-ALG-10 and COL-ALG-20 have highly interconnected networks of pores compared with ALG samples, as also reported by Govindharaj et al. [18]. Pore size significantly decreases according to the increase of marine collagen concentration while an interconnected structure useful to cell adhesion and proliferation is maintained. COL-ALG-20’s pore diameter, equal to 83 ± 27 µm, is in the range of the 20–125 μm indicated as optimal for cell attachment [43]. 

The ideal bioink should have a degradation rate equal to the new ECM deposition rate supporting in vitro skin tissue maturation [44]. The degradation profile of COL-ALG-20 is characterised by a lower degradation rate compared with COL-ALG-10 and ALG bioink with a mass remaining of 65 ± 4% after 21 days of culture resulting in an excellent structure for cell growth and tissue maturation. Govindharaj et al. showed 85% of sample degradation regardless of marine collagen concentration at 3 days [18]. The proposed formulation with 20 mg/mL of marine collagen is more stable during the in vitro culture period. In the present study, marine collagen allowed the formation of a more stable network compared to the standalone alginate bioink, as it is also confirmed by the lower swelling index that characterises both the bioink formulations prepared with the marine collagen. For the marine collagen-based formulations, the degradation is a slow process that could be offset by ECM deposition during the in vitro culture period; therefore, the effect of degradation on mechanical properties was not investigated.

As demonstrated in other studies, marine collagen has antibacterial properties even if combined with other polymers [45,46]. The marine collagen solutions at 10 and 20 mg/mL showed an antibacterial effect on *S. aureus* and *E. coli* with a more evident effect against gram positive bacteria when compared to gram negative. The same trend was observed for the COL-ALG-10 and COL-ALG-20 bioinks which have a reduced antibacterial effect only against *S. aureus.* Most likely, this result is related to the bioink crosslinking immediately after printing that enables it to obtain a more stable structure, but the crosslinking could reduce the marine collagen diffusion within the agar compared to the collagen solution.

Considering the better printability properties, the lowest in vitro biodegradation and swelling index, as well as the better antimicrobial properties the bioink formulation selected for the skin model biofabrication, was the COL-ALG-20. 

In vitro skin model represents an alternative to in vivo or ex vivo animal skin experiments. There is great interest in the development of human skin models due to the structural differences between animal and human skin tissue in terms of morphology and function, but also due to European banning of cosmetic products testing on animal models [23]. In addition, skin models could be useful for studying skin development and diseases [47]. The skin model, proposed in the present study, has a bilayered structure with human fibroblast cells for the dermal layer and human keratinocytes for the epidermal one, as most of the studies reported in the literature. The cell-laden structures were cultured in vitro for 14 days, showing NHDF fibroblast and HaCaT keratinocyte proliferation within the structure, as demonstrated by increments of cell viability measured through the XTT assay. Moreover, the live/dead assay showed, at day 14 of culture, spreading fibroblast and keratinocyte clusters of incremented diameters compared to days 1 and 7.

Few studies reported the keratinocyte-laden bioink printing for the epidermal layer biofabrication [48,49]. Lameirinhas et al. investigated the viability of 3D bioprinted construct using nanofibrillated cellulose/gellan gum hydrogel-based bioinks loaded with HaCaT cell [49]. At day 7 of culture, they observed an increase of HaCaT viability compared to day 1, but the cells in the construct were still single cells. For the bioink proposed in this study, HaCaT clusters have been observed at day 7.

Piola et al. proposed gelatin/xanthan-gum-based hydrogels for human skin cells by 3D bioprinting, demonstrating the biocompatibility and also the encapsulated fibroblast and keratinocyte proliferation [48]. Their results showed cell proliferation until day 14 of culture, as also reported in this study.

The 3D bioprinter skin model was further investigated through gene expression analysis at days 1, 7 and 14. Skin ECM has an organised structure composed of structural proteins, collagen, laminins, elastin, fibronectins, proteoglycans and hyaluronan that plays critical roles such as supporting and maintaining the integrity of the derma [50]. Hence, we analysed some ECM components, such as collagen I, collagen III, elastin and fibronectin synthesised by fibroblasts during the wound-healing process, to evaluate if the bioink was able to support ECM deposition by NDHF cells, as previously reported [30]. Regarding keratinocytes, cytokeratins have been widely used as marker proteins for various epithelial cell proliferation states. In particular, cytokeratin 6 was selected as a marker of cell–cell and cell–matrix contact [51]. Overall, the increment of gene expression observed in this study clearly highlighted the synthesis of ECM proteins in the constructs according to viability and proliferation results. However, the gene expression analysis allowed the RNA detection but the ECM deposition and keratinocytes differentiation should be deeply investigated using immunohistochemistry analysis for a qualitative evaluation of protein expression.

The histological analysis showed that the cell-laden bioink maintains the printability characteristics, allowing a bilayered structure bioprinting with two distinct layers and, according to live/dead staining, the H&E showed an increase of fibroblast number and HaCaT clusters. All together, these findings demonstrated an increase in cell proliferation and viability into the 3D structure up to 14 days. Ramakrishnan et al., in their study, constructed biofabricated skin tissue using human primary fibroblast and keratinocyte-laden bioink. These authors observed a complete multilayer of keratinocytes by H&E staining 14 days of ALI culture after 5 days of submerged culture, which corresponds to about three weeks of culture period [28]. All the studies with a native skin-like structure with complete epithelium stratification reported a culture period of at least 28 days. A longer culture period of at least 4 weeks could probably allow a complete epidermal cell stratification as well for the bioprinted construct proposed in this study.

The proposed 3D construct was investigated as an in vitro skin model; however, it could be translated in vivo for chronic skin wounds treatment. Chronic wounds represent an extreme environment with pH and temperature variations; therefore, to evaluate the possible in vivo application, the effect of these parameters should be considered. In conclusion, the obtained results highlight COL-ALG-20 as a bioink with excellent properties for potential application in the bioprinting of a skin model to use for in vitro studies. In particular, the obtained bioink can be printed in 3D structures and is able to support fibroblast and keratinocyte viability and proliferation.

## Figures and Tables

**Table 1:** Bioink formulations.

Bioink	Alginate [%]	Marine Collagen [mg/mL]	CaCl_2_ [mM]
ALG	6	-	25
COL-ALG-10	6	10	25
COL-ALG-20	6	20	25

**Table 2:** Parameters for printability assessment.

Bioink	ALG	COL-ALG-10	COL-ALG-20
Extrusion Pressure (kPa)	18	20	23
Spreading Ratio	1.61 ± 0.31	1.37 ± 0.12	1.16 ± 0.15
Shape Fidelity 4 layers	0.9 ± 0.03	0.93 ± 0.01	0.9 ± 0.02
Shape Fidelity 8 layers	0.9 ± 0.02	0.99 ± 0.03	0.98 ± 0.04

**Table 3:** Inhibition zone diameters for antibacterial activity assessment.

		Dimeter of Inhibition Zone [mm]			
		Marine Collagen Solutions		Bioinks	
		10 mg/mL	20 mg/mL	COL-ALG-10	COL-ALG-20
*S. aureus*	10^6^ CFU	40 ± 3	38 ± 1	24 ± 1	33 ± 1
	10^7^ CFU	37 ± 1	36 ± 1	18 ± 1	32 ± 1
*E. coli*	10^6^ CFU	14 ± 1	20 ± 1	-	-
	10^7^ CFU	15 ± 1	18 ± 4	-	-

## Data Availability

All data relevant to the publication are included.

## Supplementary Materials

The following supporting information can be downloaded at: https://www.mdpi.com/article/10.3390/pharmaceutics15051331/s1. Table S1: Primer sequence of the analyzed gene.
