# Supplementary material for: Marine Collagen-Based Bioink for 3D Bioprinting of a Bilayered Skin Model
Source: Pharmaceutics. 2023 Apr 24;15(5):1331. doi: 10.3390/pharmaceutics15051331 (PMC10223413; doi:10.3390/pharmaceutics15051331)
Supplement: Supplementary file 1 [file pharmaceutics-15-01331-s001.zip › pharmaceutics-2340394-supplementary.pdf]

Supplementary Materials

Table S1. Primer sequence of the analyzed gene.

GENES	***PRIMER SEQUENCE***
β-Actin	*F*-TCTGGCACCACACCT
β-Actin	*R*-TGATCTGGGTCATCT
Collagen I	*F*-GGGAACGCGTGTCAATCC
Collagen I	*R*-CAGTTACACAAGGAACAGAACAGTCTCT
Collagen III	*F*-TGGTCAGTCCTATGCGGATAGA
Collagen III	*R*-CGGATCCTGAGTCACAGACACA
Fibronectin	*F*-TCGCCATCAGTAGAAGGTAGCA
Fibronectin	*R*-TGTTATACTGAACACCAGGTTGCAA
Elastin	*F*-CTAAATACGGTGCTGCTGGC
Elastin	*R*-CATGGGATGGGGTTACAAAG
K6a	*F*-GGCTGAGGAGCGGCGTGAACAG
K6a	*R*-AAGGAGGCAAACTTGTTGTTGAG
